# Oxidative costs of reproduction in mouse strains selected for different levels of food intake and which differ in reproductive performance

**DOI:** 10.1038/srep36353

**Published:** 2016-11-14

**Authors:** Aqeel H. Al Jothery, Lobke M. Vaanholt, Nimesh Mody, Anis Arnous, Jens Lykkesfeldt, Lutz Bünger, William G. Hill, Sharon E. Mitchell, David B. Allison, John R. Speakman

**Affiliations:** 1 Institute of Biological and Environmental Sciences, University of Aberdeen, Aberdeen AB24 2TZ, UK; 2 Department of Physiology and Pharmacology, College of Veterinary Medicine, University of Karbala, Karbala, Iraq; 3 Institute of Medical Sciences, University of Aberdeen, College of Life Sciences and Medicine, Foresterhill Health Campus, Aberdeen, United Kingdom; 4 Section of Experimental Animal Models, Faculty of Health & Medical Sciences,University of Copenhagen, Denmark; 5 Animal and Veterinary Science Group, Scotland’s Rural College (SRUC), Edinburgh EH9 3JG, UK; 6 Institute of Evolutionary Biology, University of Edinburgh, Edinburgh EH9 3JT, UK; 7 School of Public Health, University of Alabama at Birmingham, Birmingham, Alabama, USA; 8 Institute of Genetics and Developmental Biology, State Key Laboratory of Molecular Developmental Biology, Chinese Academy of Sciences, Beijing, People’s Republic of China

## Abstract

Oxidative damage caused by reactive oxygen species has been hypothesised to underpin the trade-off between reproduction and somatic maintenance, i.e., the life-history-oxidative stress theory. Previous tests of this hypothesis have proved equivocal, and it has been suggested that the variation in responses may be related to the tissues measured. Here, we measured oxidative damage (protein carbonyls, 8-OHdG) and antioxidant protection (enzymatic antioxidant activity and serum antioxidant capacity) in multiple tissues of reproductive (R) and non-reproductive (N) mice from two mouse strains selectively bred for high (H) or low (L) food intake, which differ in their reproductive performance, i.e., H mice have increased milk energy output (MEO) and wean larger pups. Levels of oxidative damage were unchanged (liver) or reduced (brain and serum) in R versus N mice, and no differences in multiple measures of oxidative protection were found between H and L mice in liver (except for Glutathione Peroxidase), brain or mammary glands. Also, there were no associations between an individual’s energetic investment (e.g., MEO) and most of the oxidative stress measures detected in various tissues. These data are inconsistent with the oxidative stress theory, but were more supportive of, but not completely consistent, with the ‘oxidative shielding’ hypothesis.

Trade-offs between life history components form the basis of our understanding of the evolution of life histories^1^,^2^. It is assumed that resources (or the ability to utilise them^3^) are limited and consequently animals cannot maximize all their life history components at the same time. That is an increase in current reproductive effort comes at the cost of reduced future reproductive effort and/or increased mortality^2^. Oxidative stress has been proposed to be a potential proximal physiological cost of reproduction^4^,^5^,^6^. Oxidative stress results from an imbalance between the production of reactive oxygen species (ROS) and the capacities of antioxidants and repair systems, leading to damage to lipids, proteins, and DNA^7^. ROS include free radicals and non-radical species derived from oxygen primarily as by-products of oxidative phosphorylation in mitochondria. According to the expectations derived from the oxidative stress theory, investment in protection and repair are reduced during reproduction, leading to an increase in oxidative damage and hence leading to negative effects on future survival and fecundity.

Over the last decade many studies have attempted to evaluate this idea by measuring components of the repair and protection systems, and levels of oxidative damage that are subsequent of reproductive attempts. The resultant data, however, have proved confusing with some studies supporting the hypothesis (increased damage due to reproduction), other studies more neutral (no effect of reproduction) and some studies showing trends in the opposite direction to that predicted (reduced damage)^8^,^9^,^10^,^11^,^12^,^13^,^14^,^15^. The cause of the variation in responses has been the subject of much recent speculation^5^,^6^,^16^,^17^, including the suggestion that it may stem from poor experimental design, or differences between the field and laboratory in the extent of resource limitation. In part the variation may arise from the wide diversity of tissues and assays that have been measured in different studies. In general, measurements based on serum or plasma have been supportive of the hypothesis, but measurements on tissues have been less supportive or contradictory^17^. Indeed recent studies have shown that it is possible to get data both confirming and refuting the hypothesis in the same individuals by examining responses in different tissues, or using different markers^18^,^19^. This raises the question of what tissues and biomarkers provide the most critical tests of the hypothesis^17^, and why different tissues show such variable responses. It has been suggested that one reason why females in lactation may show a reduced level of damage in some tissues is because there are trans-generational consequences of the levels of damage sustained by the female during reproduction^20^. Females may therefore protect themselves during reproduction because this shields their offspring from oxidative damage: the ‘oxidative shielding hypothesis’^20^. The tissues where damage is generally shown to be reduced (the liver) may be crucial in this respect because some of the secreted substrates in milk may be synthesised in the liver. Lactating females may therefore protect their livers (and presumably their mammary glands) to shield their offspring, but in so doing neglect to protect other tissues that would likely not impact their offspring, but have negative life history consequences for themselves.

One tissue that may be crucial in this respect is the brain. The brain is post-mitotic and has been shown to be highly susceptible to oxidative damage. Paradoxically, however, very few studies have measured the consequences of reproduction on oxidative damage in the brain, one of which concerned male rats where costs of reproduction are low^21^, and two others concerned lactating striped hamsters and lactating mice that showed opposing results, with increased oxidative damage in brains of lactating hamsters (measured by hydrogen peroxide)^22^ and reduced damage in lactating mice (measured by protein carbonyls) compared to non-reproductive controls^23^. In the current study we measured the consequences of reproduction (lactation) in female mice for oxidative damage in a liver, brain and mammary tissue. We used a variety of biomarkers of oxidative protection (dROMS and antioxidant enzyme activities of SOD; superoxide dismutase, GPx; glutathione peroxidas and CAT; catalase) and damage (protein carbonyls and 8OHdG), and compared these in lactating and non-reproducing mice from two strains selected for different levels of food intake (high, H and low, L). We have previously shown that these lines differ significantly in their levels of reproductive performance with H mice having a higher performance than L mice, i.e., milk production was greater and weaned pups that were ~30% larger^24^. In line with the oxidative stress theory, we hypothesised that mice from the H line, with their higher reproductive effort, would reduce investment in protection and repair during lactation, leading to an increase in oxidative damage compared to L mice. Similarly, lactating mice would be expected to have increased damage compared to non-reproducing mice.

## Results

### Selection lines

Reproductive success was higher in H mice than in L mice with 22 out of 29 vs. 15 out of 41 litters successfully weaning pups. Body mass (BM) prior to mating did not differ between mice that did or did not fall pregnant within each line, i.e., for L line mean BM of non-pregnant mice and pregnant mice was 27.0 ± 2.6 and 25.6 ± 2.16 respectively (t-test: p = 0.08) and for H line it was 25.5±3.1 and 24.9 ± 2.0 respectively (t-test: p = 0.6). Also, there were no significant differences in BM between mice of the L and H line prior to mating (Table 1).

During lactation H mice had 62% higher metabolisable energy intake (MEI) and more than doubled milk energy output (MEO) compared to L mice. H mice weaned 25% larger pups (Table 1).

### Serum

#### Effects of line and reproductive status

**Figure 1:**
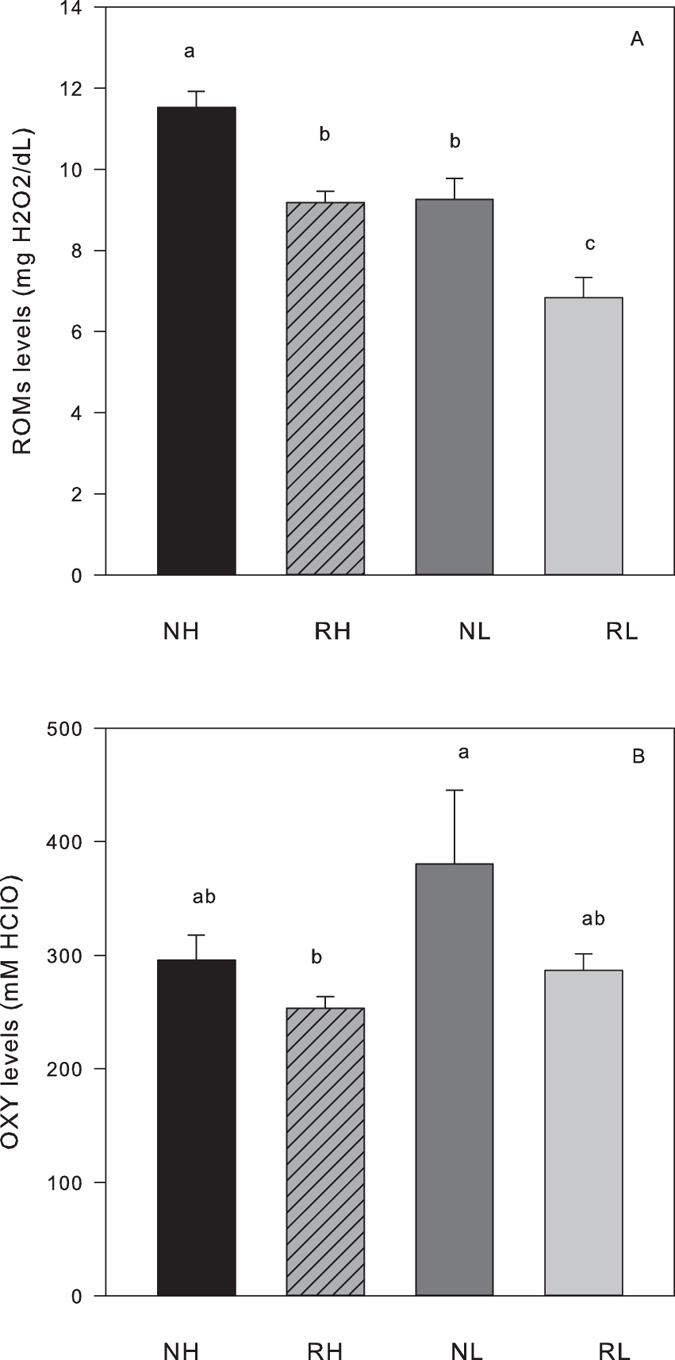
Markers of oxidative stress in the serum of non-reproducing female mice (NH, black bars, N = 10; NL, dark grey bars, N = 10) and reproducing mice at day of weaning (RH, striated bars; N = 20, RL, light grey bars, N = 14) (**A**) Reactive oxygen metabolites (ROMs). (**B**) Total non-enzymatic antioxidants capacity (OXY, untransformed data). Different letters inside the bars indicate significant differences between groups.

General linear models (GLM) with reproductive status (non-reproductive, N vs. reproductive, R) and line (H vs. L) as fixed factors were performed to test for differences between oxidative stress markers in serum and tissues. Reactive oxygen metabolites (dROM) and non-enzymatic antioxidant activity (OXY) levels in serum were reduced by 15–25% in reproductive (R) versus non-reproductive (N) mice (Table 2 and Fig. 1). In addition, H mice had significantly higher levels of dROMs than L mice of both reproductive groups.(Table 2, Fig. 1A). A significant effect of line was also found for levels of OXY, but post-hoc Tukey tests did not indicate any differences between RH and RL mice or NH and NL mice (Table 1, Fig. 1B). The serum oxidative stress index (OSI = dROM/OXY*1000) was not significantly different between R and NR, but did differ significantly between the lines, with H mice having a higher OSI compared to L mice by approximately 25%.

##### Effect of body mass, food intake and reproductive traits

General linear models (GLM) were rerun with several individual variables (i.e., BM, food intake; FI, and the reproductive traits: metabolisable energy intake; MEO, daily energy expenditure; DEE, litter mass; LM or litter size; LS) as covariate to test how these variables affected the results. No significant correlations with BM, food intake (FI) or reproductive traits and these oxidative stress biomarkers were observed in General Linear Models (GLM) where they were added as covariates (See Supplementary Table 1&2).

### Liver

#### Effects of line and reproductive status

**Figure 2:**
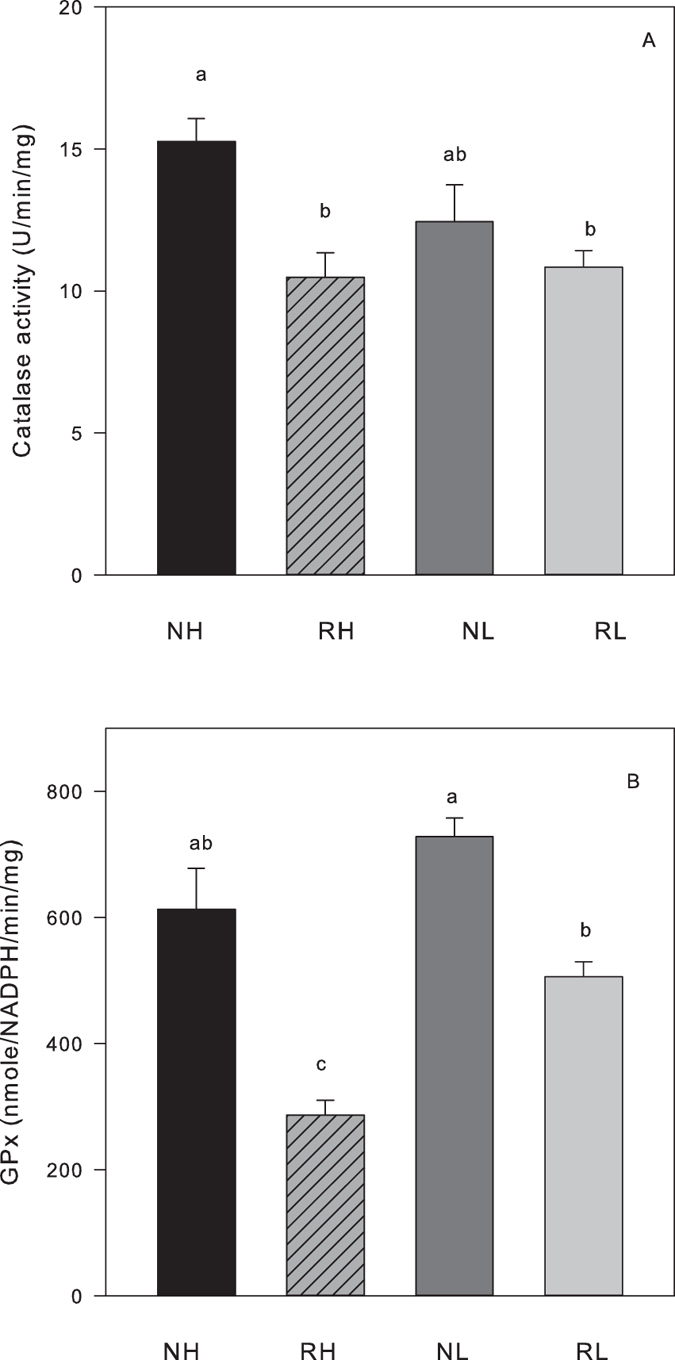
Markers of enzymatic antioxidants in the liver of non-reproducing female mice (NH, black bars; NL, dark grey bars) and reproducing mice at day of weaning (RH, striated bars; RL, light grey bars). (**A**) Catalase (CAT) activity. (**B**) Glutathione peroxidase (GPx) activity. Different letters inside the bars indicate significant differences between groups (N = 10 per group).

No significant effects of line or reproductive status were found on oxidative stress markers measured in liver, except for the antioxidants catalase (CAT) and Glutathione Peroxidase (GPx) (Table 3). Both, CAT and GPx activity were significantly lower in reproductive compared to non-reproductive mice (Table 3, Fig. 2A). In addition, a significant effect of selection line was found for GPx, with lower levels observed in H compared to L mice (Table 3, Fig. 2B). Post-hoc Tukey tests revealed a significant difference between H and L mice in reproductive, but not in non-reproductive mice (Fig. 2B).

##### Effect of body mass, food intake and reproductive traits

**Figure 3:**
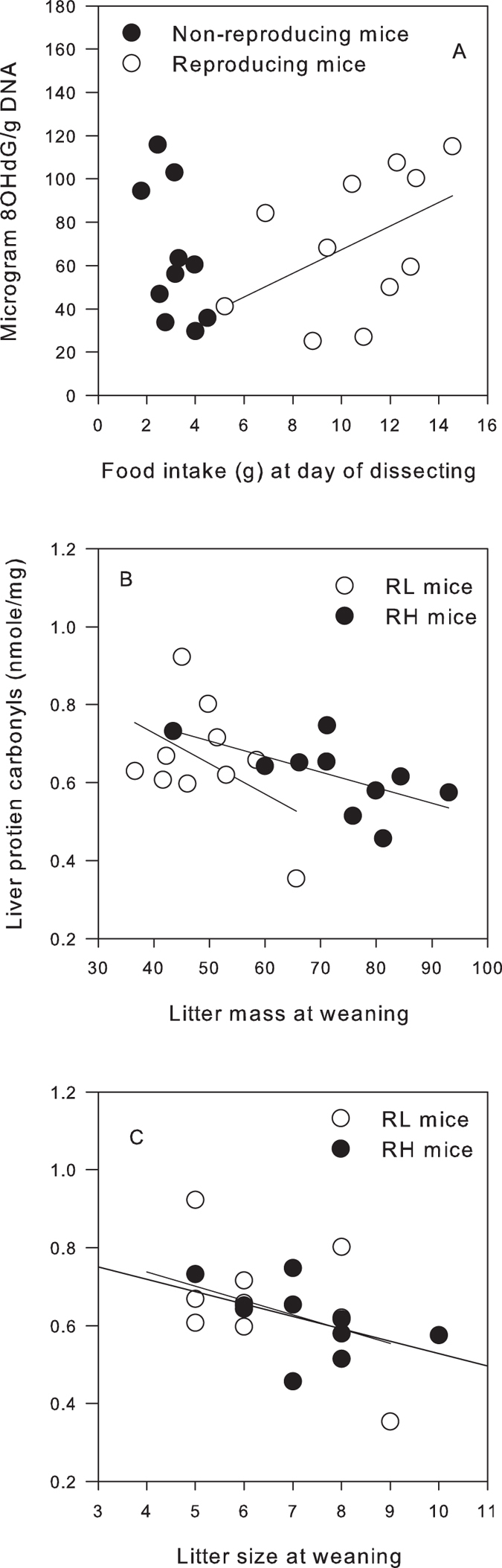
Pearson’s correlations between (**A**) Food intake and DNA damage (measured by ELISA) in pooled reproducing mice (RH and RL, *N* = 11, filled circles) and pooled non-reproducing mice (NH and NL, *N* = 10, open circles). Reproducing mice, R^2^ = 0.473, P-Value = 0.142; Non-reproducing mice, R^2^ = −0.559, P-Value = 0.093 (**B**) Litter mass and liver protein damage (PC) in reproducing mice, RH mice (*N* = 10, filled circles) and RL mice (*N* = 10, open circles). Regression lines for RH mice,. R^2^ = −0.62, P-Value = 0.05. Regression line for RL mice,. R^2^ = −046, P-Value = 0.186., (**C**) Litter size and liver protein damage (PC) in reproducing mice, RH mice (*N* = 10, filled circles) and RL mice (*N* = 10, open circles). RH mice, R^2^ = 0.148, p = −0.492, RL mice, R^2^ = 0.246. p = − 0.404.

An inverse relationship between BM and protein carbonyl (PC) levels was found and this relationship did not differ with line or reproductive status (Supplementary Table 1, GLM with BM as covariate, line; F_1,36_ = 2.42, p = 0.13, Reproductive status, F_1,36_ = 0.01, p = 0.92, BM, p = 0.03, y = −0.1x + 3.09). FI and measurements of reproductive traits (MEI, DEE, and MEO) were not significant covariates in GLMs for any measure of liver oxidative stress (Supplementary Table 1&2). Although, a significant interaction between MEI and line was found for both superoxide dismutase (SOD) and GPx activities (SOD, MEI× line, *P* = 0.02; GPx, MEI× line, *P* = 0.03). Pearson correlation showed that both SOD and GPx activity in reproductive L mice were positively correlated to MEI (SOD, *r* = 0.65, *P* = 0.04; GPx, *r* = 0.67, *P* = 0.05), whereas this correlation was not significant in reproductive H mice (P > 0.05). Additional markers of reproductive effort (LM and LS) were inversely related to protein damage in both lines (Pearson correlations: LM, *r* = −0.45, *P* = 0.048, Fig. 3C; LS, *r* = −0.46, *P* = 0.043; Fig. 3D). Mice with higher reproductive effort (heavier LM or larger LS) had reduced protein damage in their liver. No significant associations between reproductive effort and any other measures of liver oxidative stress were found (all *P* > 0.05, See Supplementary Table 2).

### Brain

#### Effects of line and reproductive status

**Figure 4:**
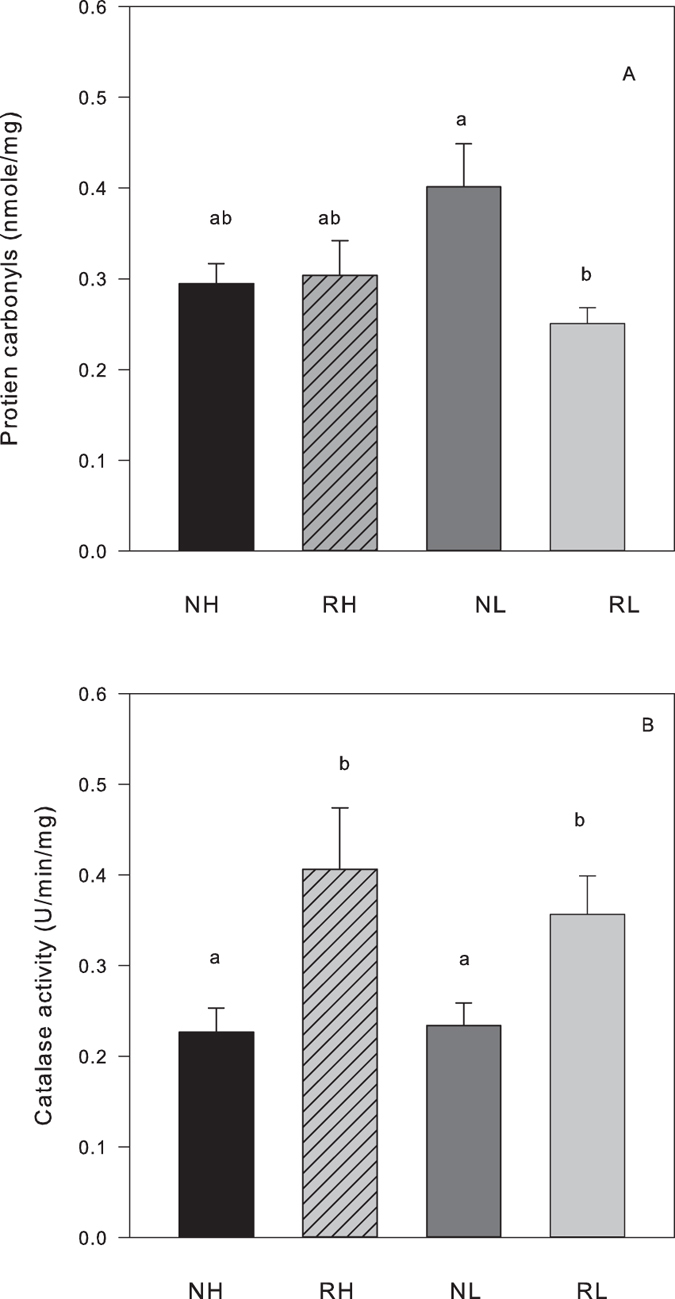
Markers of oxidative damage and enzymatic antioxidant in the brain of non-reproducing female mice (NH, black bars; NL, dark grey bars) and reproducing mice at day of weaning (RH, striated bars; RL, light grey bars, N = 10 for each group). (**A**) Protein carbonyls (PC, untransformed data). (**B**) Catalase (CAT, untransformed data) activity. Different letters inside the bars indicate significant differences.

Levels of PC were significantly decreased in reproductive vs non-reproductive mice and levels of CAT were significantly increased, but no effects of line were observed (Table 4, Fig. 4). The other oxidative stress markers were not affected by reproductive status or line (Table 4).

##### Effect of body mass, food intake and reproductive traits

Despite the lack of differences between the treatment groups, there was a significant interaction between line and BM for SOD activity indicating that the relationship between SOD and BM differed between the lines (Line x BM; F_1,35_ = 5.01, P = 0.03, Supplementary Table 1). Indeed, SOD activity in non-reproductive H mice was positively correlated with BM (y = 1, 71x − 31.5, *r* = 0.66, *P* = 0.05), but this relationship was absent in non-reproductive L mice (r = −0.47, P = 0.17) and reproductive H (r = 0.47, P = 0.18) and L mice (r = −0.08, P = 0.82). FI and BM were not significant covariates in GLMs with any other oxidative stress biomarkers in the brain. GLMs with reproductive traits revealed a significant interaction between line and DEE for GPx activity (Line x DEE; F_1,14_ = 4.71, P = 0.04)); i.e., GPx activity was inversely correlated with DEE in reproductive L mice (y = 3.32x + 289, *r* = −0.72, *P* = 0.03), but not in reproductive H mice (*r* = 0.12, *P* = 0.76, see Supplementary Table 2).

### Mammary glands

#### Effects of line and reproductive status

No significant differences between lactating mice of the two strains (H and L) were found for any of the measured biomarkers of oxidative stress in mammary tissue (Table 5).

##### Effect of body mass, food intake and reproductive traits

Food intake was negatively correlated with mammary gland GPx activity (GLM, *F*_1,32_ = 4.9, *P* = 0.03). Correlation between reproductive traits and oxidative stress measures in the mammary gland revealed a significant interaction between line and LS for SOD activity (GLM, *F*_1,32_ = 4.9, *P* = 0.03), but the correlation between SOD activity and LS did not reach significance in either line (RH; *r* = 0.38, *P* = 0.08, RL; *r* = −0.36, *P* = 0.21, Supplementary Table 2).

## Discussion

We measured a variety of biomarkers of oxidative stress, and compared these in lactating (R) and non-reproducing mice from two strains that had been selected for different levels of FI, that we have previously shown differ significantly in their levels of reproductive performance^24^.

As in previous studies, there were no differences in BM between mice from the L and H strain prior to mating, but reproductive success was increased in mice of the H line^24^. That is mice from the H line produced larger litters and weaned larger pups. This can be attributed to the increased MEI and MEO in H mice resulting in higher energy availability for pup growth. The oxidative stress theory predicts that there is a trade-off between reproduction and somatic maintenance^4^,^5^,^6^ and mice from the H line, with their higher reproductive effort, would thus be expected to reduce investment in protection and repair during lactation, leading to an increase in oxidative damage. Similarly, mice that were enabled to reproduce would be expected to have increased damage compared to mice that were prevented from reproducing. In agreement, with this prediction antioxidant protection measured by OXY levels in serum, and CAT and GPx activity in livers of the lactating mice were reduced compared to non-reproductive mice. However, oxidative damage (measured by dROMs in serum, PC in the liver and brain and 8OHdG in the liver), which would be anticipated to be increased, was unaltered or reduced in lactating mice. Also, when comparing mice with different levels of reproductive effort (H vs. L mice) contradictory results were found, with no differences in antioxidant protection in serum, liver, brain or mammary tissue (except for GPx in liver which was reduced in H mice) and no differences in oxidative damage, except in serum (dROMs), where reproductive L mice were found to have reduced damage.

Previous studies in both wild and laboratory animals studying the relationship between reproductive effort and oxidative stress have shown similarly ambiguous results. Several studies have shown oxidative damage (dROMs) to be unchanged in reproductive vs. non reproductive animals^11^,^12^,^13^,^15^,^25^,^26^, whereas others have found a positive association between lactation and oxidative damage in serum^9^,^10^,^27^. In addition, within the same species contradictary results have been reported showing increased oxidative damage in one macromolecule (proteins), but not in others (lipids and DNA)^18^,^19^. Several studies in mammals and birds have found oxidative damage to be positively related with natural LS/clutch size (Mammals:^9^,^10^, Birds:^28^,^29^). However, the present and other studies in mammals and birds found no such relationship^15^,^22^,^30^,^31^,^32^,^33^ or a negative relationship^31^. Also, house mice with increased LS did not show any changes in enzymatic antioxidants (glutathione, oxidised glutathione, and CAT) in various tissues (liver, heart, muscle) compared to mice with reduced LS^34^. Studies in mice with increased LS and concurrent pregnancy did not show increased oxidative damage (glutathione and protein thiol) in the liver, heart and urine (8OHdG) compared to those with increased litters^35^.

These findings are mostly inconsistent with the predictions from the oxidative stress theory and are in agreement with the idea that antioxidant levels are responsive to ROS production, and hence low levels of antioxidants do not necessarily translate into increased oxidative damage^7^,^36^,^37^. Changes in enzymatic antioxidant levels for any given condition may depend on the capacity of a steady-state level of tissue antioxidant and on the particular sites where ROS are generated. Since SOD is the first enzyme acting on the main superoxide free radical produced from the electron transport chain, converting it to H_2_O_2_^38^, the steady-state level of SOD in liver may be sufficient to cope with any elevation in O- production. This might explain why we did not observe a change in SOD activity between reproductive and non-reproductive mice. Given the main role of CAT and GPx activity to convert H_2_O_2_ to water^39^, the observed reduction of liver CAT and GPx activity in lactating mice with no associated oxidative damage to protein and DNA (specifically induced by OH^−^ that derived from H_2_O_2_) suggests that the levels of H_2_O_2_ that needed to be detoxified were actually lower in lactation. This could be linked to an increased metabolic rate^33^,^40^. Since other important antioxidants (e.g. non-enzymatic and exogenous antioxidants) were not measured here, it is possible that these levels may be responsible for the lack of increased damage in the liver tissue.

Contrary to the expectations from the oxidative stress theory a reduction in or lack of oxidative damage in reproductive mice compared to non-reproductive mice was found in this study, as has been observed many times previously^9^,^12^,^14^,^23^. The oxidative shielding hypothesis proposed by Blount *et al*.^20^ hypothesises that transition to the reproductive state triggers a pre-emptive reduction in levels of oxidative damage in certain markers and tissues, to shield mothers, and in particular their gametes and developing offspring, from harm caused by an inevitable increase in oxidative damage resulting from the increased expenditure of reproductive effort. This would explain why in studies comparing reproductive vs. non-reproductive animals a reduction in oxidative damage is often observed.

Another explanation for a lack of oxidative damage in reproductive mice stems from the fact that reproductive mice increase their FI many-fold during lactation; it has been hypothesised that an increase in the gross intake of dietary antioxidants - as a direct consequence of increased FI during lactation - may result in reduced oxidative damage in laboratory animals^12^. If this was correct a positive relationship would be expected between food consumption and antioxidant levels in the serum^8^,^10^. However, the lack of association between the total antioxidants in the serum and food intake in the present study, combined with the reduction of total antioxidants in reproductive compared to non-reproductive mice suggested the reduction of oxidative damage observed in lactating mice was unlikely to be driven by the difference in oral antioxidant consumption. In agreement with this, a recent study has shown no differences in oxidative damage between lactating mice fed standard or low antioxidant diets^23^.

Lactation is recognised as the most energetically demanding phase of a mammal’s life^1^. On the day of dissection, MEO of reproductive mice in both lines was almost tripled compared to non-reproductive mice, but no corresponding increase in oxidative damage was observed, confirming that a simple link between expenditure and ROS production is too simplistic (see also^33^,^40^). In addition, GLMs with DEE as a covariate revealed no significant relationships between DEE and any of the oxidative stress markers measured. Previous work on the relationship between energy expenditure and oxidative protection and damage is confused. Experimentally increased energy demands, by exposure to the cold, produced complex patterns of change in protection and damage in short-tailed field voles (Microtus agrestis)^41^,^42^,^43^ and mice^44^ with no overall effect on survival^43^,^44^. In contrast, Fletcher et al.^10^ did find a positive relationship between DEE and plasma oxidative damage (protein carbonyls) in lactating red squirrels.

The current study is one of the first to study impacts of reproduction on oxidative stress to the brain. The brain may be an important target for oxidative damage because it is enriched in polyunsaturated fatty acids which are prone to be oxidised^45^. In addition, brain tissue is recognised to have a low antioxidant capacity combined with a high iron metal content^46^. Finally, it is widely thought that post-mitotic tissues like the brain are more likely to accumulate oxidative stress due its slower turnover rate^47^. A recent study of the responses to oxidative stress in greenfinches that had been fed paraquat showed that mortality risk was only correlated with damage to brain tissue^48^. In contrast with the predictions of life history –oxidative stress theory, the lactating mice observed here and in another study on lactating MF1 mice^23^ had significantly lower brain protein damage compared to NR mice. This result is inconsistent with work on lactating striped hamsters where ROS production, measured by hydrogen peroxide, was significantly increased in lactating hamsters compared to NR hamsters^22^. Although, in the same study there was no change in the brain damage measured by MDA^22^. This again points out the difficulties in comparing studies that use different biomarkers for oxidative damage or in different species. However, caution is needed in interpreting the data on oxidative stress in whole brain tissue as different parts of the brain may be affected differently^49^,^50^. The observed reduction in brain oxidative damage is consistent with the elevation of brain CAT activity and may be consistent with the oxidative shielding hypothesis. Although it is unlikely that damage to the female’s brain could directly impact the offspring in the same way that damage in the liver, serum and mammary glands could, one could speculate that it is easier to activate protective mechanisms all over, rather than switching it on in specific tissues and not in others. If this is the case, however, one would have expected to detect a decrease in oxidative damage in both the liver and mammary tissue as well, which was not observed here. We actually showed a reduction in protein damage in liver with increased reproductive effort (i.e., LS) which contradicts the predictions from the empirical data and oxidative shielding hypothesis where a positive relationship between reproductive effort and oxidative damage is expected^20^.

There is a considerable literature suggesting that breastfeeding in humans reduces breast cancer risk^51^. Moreover, human breast cancer risk was inversely related to the number of children that had been breast-fed over an individual’s life^52^. It would hence be expected that oxidative stress would be lower among lactating mice with larger litter sizes. However, consistent with the results of the only other study investigating oxidative stress in mammary tissue of mice^53^, we found that multiple measures of oxidative stress in the mammary gland were not significantly altered between mice with different reproductive effort (H vs. L mice). Hadsell et al.^53^ measured the levels of oxidative damage to proteins and DNA in the mammary gland of mice during a normal lactation relative to those mice forced to prolong lactation. Mitochondrial protein and DNA damage markers (PC and 8OHdG) were significantly increased for mice forced to prolong lactation. However, oxidative damage markers (PC and MDA) in the whole mammary homogenate were not significantly different between the groups^53^. The paucity of studies on the mammary gland in small rodents may be because mammary tissue may transdifferentiate to adipose tissue when animals are not breeding^54^ and hence there is no available comparison tissue in the non-breeding animal, although comparisons between individuals with different levels of reproductive performance, like we did here, can be made.

The lack of signs of oxidative stress changes caused by reproduction in the mammary gland could possibly be due to high levels of protein turnover in these tissues^47^. Daily protein turnover in the mammary glands of lactating rats was shown to be around 62%^55^. The inverse relationship observed between food intake in lactation and GPx activity, with no increased protein damage, suggested that other important antioxidant systems (e.g. non-enzymatic antioxidants) may be responsible for the lack of increased damage in the mammary gland.

## Conclusions

The present study showed unchanged (liver) or reduced (brain and serum) levels of oxidative damage in lactating versus non-lactating mice. Moreover, no differences in multiple measures of oxidative stress in the liver (except for GPx), brain and mammary glands were observed between mice with different levels of reproductive effort (H vs. L lines). Associations between measures of oxidative damage and reproductive performance demonstrated that oxidative damage (protein carbonyls) was reduced with greater reproductive effort (liver), Finally, there were no associations between the individual’s energetic investment (MEI, DEE, MEO) and almost all of the oxidative stress measures detected in various tissues.

These findings are inconsistent with the life history-oxidative stress theory which predicts that oxidative damage should be higher among lactating mice and with increased reproductive effort. The reduction in oxidative damage in the brain of reproductive animals and the absence of an increase in damage to the mammary tissue during reproduction is consistent with predictions from the oxidative shielding hypothesis^20^. However, the observed negative association between oxidative damage in the liver and reproductive effort was not. At present there is no comprehensive idea explaining the totality of the available data^6^,^17^.

## Materials and Methods

### Source of mouse lines

We used mice, *Mus musculus*, from the maintenance (M) requirement strains^56^ which originated from a common background generated by a three strain cross, which were selected for 38 generations for high (H) and low (L) food intake (FI, for more details see^57^). We have recently shown that a correlated trait to the selection on FI is a large difference in reproductive performance^24^, with the high intake line having much greater reproductive investment than the low intake line.

### Breeding protocol and sample collection

Virgin female mice, aged 9–12 weeks, were individually housed in shoebox cages (48 cm × 15 cm × 13 cm) under a 12 h L: 12 h D photoperiod at 21 ± 2 °C and a relative humidity of 59 ± 5%. All cages were provided with sawdust, paper bedding and a cardboard tube. Animals had *ad libitum* access to water and food (D12450B, Research Diets, New Brunswick, NJ, USA). After 12 days of baseline monitoring, seventy females (*N* = 29 for RH and *N* = 41 for RL) were mated to non-sibling males of the same line for 11–15 days. Twenty age-matched adult females (*N* = 10 for each line) were not mated and considered as non-reproductive (N) controls (NH & NL). Pregnant mice (R, *N* = 22 for RH and *N* = 15 for RL) were monitored daily to establish the day of parturition (day 0), and the timing for pregnancy was back calculated from the day of birth as day −1 (last day of pregnancy) to day −18 (beginning of pregnancy). FI, body mass (BM), litter size (LS), litter mass (LM) and pup mass (PM = LM/LS) for lactating mice and their pups were measured from day 5 until day 18 of lactation. Lactation was terminated on day 18 and all mice were culled by CO_2_ overdose. Blood samples were collected into non-heparinised tubes by cardiac puncture and left on wet ice to clot. Serum was extracted using centrifugation at 14000 g for 10 minutes at 4 °C and stored at −80 °C for later analysis. Liver, brain and mammary tissue (for reproductive mice, R only) were collected from all animals and stored at −80 °C until analysis.

All procedures concerning animal care and treatment were carried out in accordance with the protocols approved by the ethical committee for the use of experimental animals of the University of Aberdeen, and were licensed by the UK Home Office under PPL 60/3705.

### Metabolisable energy intake (MEI), Daily energy expenditure (DEE) and Milk energy output (MEO)

Measurements of MEI were performed on days 12–14 of lactation and the doubly labelled water (DLW) technique was used to measure DEE over days 15–17 of lactation. MEI values were then subtracted from the estimated DEE to calculate MEO (For details of the procedures see^58^.

### Reactive oxygen metabolites (ROMs) and non-enzymatic antioxidant capacity (OXY)

The levels of reactive oxygen metabolites and total antioxidants (exogenous and endogenous) present in serum were quantified using the dROMS assay and OXY-Adsorbent test, respectively following the manufacturers protocol (Diacron, Grosseto, Italy). For more details see supplementary materials A.

### Enzymatic antioxidant activities

Frozen tissue samples were prepared for enzymatic activity measurements by homogenising them in 1:20 ratio of ice-cold 50 Mm phosphate buffer. After centrifugation (4000 g at 4 °C for 20 minutes), the supernatant was kept at −80 °C until analysis. Catalase (CAT) activity was measured using fresh supernatant with the end point assay specifically for tissues mentioned by Cohen et al. (1970) and further used by Aebi (1984). Total superoxide dismutase (SOD) activity was measured according to the method of Marklund and Marklund^59^. Glutathione peroxidase (GPx) activity was measured according to the method of Paglia and Valentine^60^ and followed by Lawrence and Burk^61^. For more details see supplementary materials B.

### Oxidative damage to protein (Protein carbonyls)

Protein oxidative damage in the different tissues was determined by measuring the quantity of protein carbonyls in a protein sample following derivatisation of proteins with dinitrophenylhydrazine (DNP, Protein carbonyl enzyme immune-assay kit, BIOCELL Corporation Ltd., New Zealand) as described previously^41^. Before the analysis protein concentration for each sample was determined using the Bradford assay to calculate the amount of sample required in the test (200–300 μg of protein).

### Oxidative damage to DNA

DNA damage in liver tissue was detected by measuring the levels of 8-hydroxy-2-deoxyguanosine (8-OHdG). Two different methodologies were used. First by Enzyme linked immune-sorbent assay (ELISA). Nuclear DNA was extracted from subsamples of liver tissue (Reproducing high line (RH) = 6, Reproducing low line (RL) = 5; Non-reproducing high line (NH) = 5, and non-reproducing low line (NL) = 5). Extraction, purification, and enzymatic digestion of DNA were performed as described by Huang et al^62^. 2. High-performance liquid chromatography coupled with electrochemical detector (HPLC-ECD).Subsamples of frozen liver tissue (N = 6 for each group) were analysed at the LIFEPHARM centre-University of Copenhagen. See supplementary materials C.

### Statistical Analysis

Data were tested for normality using the Shapiro-Wilkinsons test and natural logarithms were used to normalize data where required. General linear models (GLM) with line, reproductive status, and the interaction between line and reproductive status as fixed factors were used to determine whether reproductive status (R vs. N) or line (H vs. L) affected levels of the oxidative stress markers in various tissues. These models were rerun including various covariates (e.g., BM, FI or reproductive traits, i.e., LM, LS & MEO) to investigate relationships between these variables and oxidative stress markers, including interaction terms with the covariate to establish whether the relationships differed between the groups. All interactions were not significant unless otherwise stated in the results. Pearson correlations were used to determine relationships between variables within the different groups. Data are presented as means ± s.e.m. Differences were considered statistically significant at *P* < 0.05 (2-tailed). Minitab (Version 16; Minitab Inc., State College, PA, USA) was used to perform all statistical analyses.

## Additional Information

**How to cite this article**: Jothery, A. H. A. *et al*. Oxidative costs of reproduction in mouse strains selected for different levels of food intake and which differ in reproductive performance. *Sci. Rep.* **6**, 36353; doi: 10.1038/srep36353 (2016).

**Publisher’s note:** Springer Nature remains neutral with regard to jurisdictional claims in published maps and institutional affiliations.

## Supplementary Material

Supplementary Information

## Figures and Tables

**Table 1:** Reproductive performance of lactating female mice selected high (H) and low (L) food intake.

Variables	H mice(N = 22)	L mice (N = 15)	Outcomes of T-test
Body mass (g) measured prior to reproduction	24.9 ± 0.4	25.2 ± 0.6	*t*_1,26_ = −0.32, *P* = 0.75
Body mass (g), d15 of lactation	32.8 ± 0.7	29.9 ± 0.8	*t*_1,30_ = 2.77, *P* = 0.01
MEI (kJ d^−1^, d12-d14 of lactation	210.9 ± 7.6	129.4 ± 6.5	*t*_1,34_ = 8.12, *P* < 0.001
DEE (kJ d^−1^), d15-d17 of lactation	90.4.4 ± 2.	72.2 ± 2.1	*t*_1,34_ = 5.05, *P* < 0.001
MEO (kJ d^−1^)	120.6 ± 6.7	57.1 ± 6.7	*t*_1,34_ = 7.19, *P* < 0.001
Litter size at weaning (d18)	7.1 ± 0.3	5.7 ± 0.4	*t*_1,26_ = 2.48, *P* = 0.02
Litter mass (g) at weaning	70.9 ± 2.6	45.2 ± 2.8	*t*_1,32_ = 6.65, *P* < 0.001
Pup mass (g) at weaning	10.1 ± 0.2	8.1 ± 0.3	*t*_1, 26_ = 6.42, *P* < 0.001

Mean ± SEM for variables measured in H and L mice prior and during lactation. Results for independent t-test are shown. MEI: Metabolisable energy intake; DEE: Daily energy expenditure; MEO: Milk energy output. Values shown are mean ± SEM.

**Table 2:** Descriptive statistics for oxidative markers measured in serum of lactating and non-reproductive mice selected for high and low food intake.

Variables	NH	RH	NL	RL	Line	Reproductive status
n	10	20	10	14		
dROMs	11.52^a^ ± 0.40	9.18^b^ ± 0.28	9.26^b^ ± 0.52	6.83^c^ ± 0.50	*F*_1,51_ = 31.6, ***P*** **<** **0.001**	*F*_1,51_ = 31.7, ***P*** **<** **0.001**
OXY	295.90^a,b^ ± 22.0	253.30^b^ ± 10.60	380.10^a^ ± 65.40	286.90^a,b^ ± 14.20	*F*_1,51_ = 5.7, ***P*** **=** **0.020**	*F*_1,51_ = 5.5, ***P*** **=** **0.023**
OS Index	40.40^a^ ± 2.57	37.51^a^ ± 1.99	30.64^a,b^ ± 5.09	24.15^b^ ± 1.85	*F*_1,51_ = 19.1, ***P*** **<** **0.001**	*F*_1,51_ = 2.6, ***P*** **=** **0.11**

Measurements of reactive oxygen metabolites (dROMs) and total non-enzymatic antioxidants (OXY) in reproductive and non-reproductive mice of selection lines for high or low food intakes. OS index was calculated by dividing dROMS by OXY * 1000. Values shown are mean ± SEM. Results of two-way ANOVAs are shown with line and reproductive status as fixed factors. No significant interactions between line and reproductive status were found in the models and these were therefore removed before final analysis.NH: Non-reproducing high food intake mice; RH: Reproducing high food intake mice; NL: Non-reproducing low food intake mice; RL: Reproducing low food intake mice. Different letters indicate significant differences between groups (post-hoc Tukey tests, p < 0.05).

**Table 3:** Descriptive statistics for oxidative markers measured in liver tissue of lactating and non-reproductive mice selected for high and low food intake.

Variables	NH	RH	NL	RL	Line	Reproductive status	n
PC	0.64 ± 0.03	0.62 ± 0.03	0.68 ± 0.03	0.66 ± 0.05	F_1,37_ = 1.87, P = 0.180	F_1,37_ = 0.29,P = 0.592	40
8OHdG*	57.0 ± 12.90	76.10 ± 14.50	70.80 ± 15.20	63.80 ± 13.70	F_1,18_ = 0.01, P=0.990	F_1,18_ = 0.22, P = 0.647	21
8OHdG^†^	2.84 ± 0.44	1.57 0.24	2.16 ± 0.68	4.70 ± 1.03	F_1,21_ = 0.98, P = 0.333	F_1,21_ = 0.90, P = 0.354	24
SOD	12.11 ± 1.46	14.12 ± 0.83	14.19 ± 0.65	12.99± 0.97	F_1,37_ = 0.20, P = 0.654	F_1,37_ = 0.20, P = 0.710	40
CAT	15.27^a^ ± 0.81	10.48^b^ ± 0.87	12.44^a,b^ ± 1.29	10.83^b^ ± 0.60	F_1,37_=1.70, P = 0.210	F_1,37_ = 11.30, **P = 0.002**	40
GPx	612.70^a,b^ ± 65.10	286.10^c^ ± 24.09	727.50^a^ ± 29.80	506.20^b^ ± 23.10	F_1,35_ = 18.50, **P < 0.001**	F_1,35_ = 49.60, **P < 0.001**	38

Measurementsof various oxidative stress markers in liver tissue of reproductive (R) and non-reproductive (N) mice of selection lines for high (H) or low (L) food intakes.Values shown are mean ± SEM. Results of two-way ANOVAs are shown with line and reproductive status as fixed factors. No significant interactions between line and reproductive status were found in the models and these were therefore removed before final analysis. NH: Non-reproducing high food intake mice; RH: Reproducing high food intake mice; NL: Non-reproducing low food intake mice; RL: Reproducing low food intake mice. PC: Protein carbonyls; 8OHdG: 8-hydroxy-2-deoxyguanosine; *measured by ELISA method; ^†^measured by HPLC method; SOD: Superoxide dismutase; CAT: Catalase; GPx: Glutathione peroxidase. Different letters indicate significant differences between groups (post-hoc Tukey tests, p < 0.05).

**Table 4:** Descriptive statistics for oxidative markers measured in brain tissue of lactating and non-reproductive mice selected for high and low food intake.

Variables	NH	RH	NL	RL	Line	Reproductive status	n
PC	0.31^a,b^ ± 0.02	0.30^a,b^ ± 0.04	0.40^a^ ± 0.04	0.25^b^ ± 0.01	*F*_1,37_ = 0.12, *P* = 0.780	*F*_1,37_ = 4.90, ***P* = 0.032**	40
SOD	17.05 ± 2.54	17.44 ± 1.95	13.51 ± 1.21	15.50 ± 2.34	*F*_1,36_ = 1.83, *P* = 0.190	*F*_1,36_ = 0.40, *P* = 0.550	39
CAT	0.23^a^ ± 0.02	0.41^b^ ± 0.06	0.23^a^ ± 0.02	0.36^b^ ± 0.04	*F*_1,37_ = 0.001, *P* = 0.950	*F*_1,37_ = 10.9, ***P* = 0.002**	40
GPx	40.90 ± 12.20	52.30 ± 11.60	24.10 ± 4.75	51.40 ± 12.20	*F*_1,32_ = 0.62, *P* = 0.460	*F*_1,32_ = 2.70, *P* = 0.110	35

Measurements of various oxidative stress markers in brain tissue of reproductive (R) and non-reproductive (N) mice of selection lines for high (H) or low (L) food intakes. Values shown are mean ± SEM. Results of two-way ANOVAs are shown with line and reproductive status as fixed factors. No significant interactions between line and reproductive status were found in the models and these were therefore removed before final analysis. NH: Non-reproducing high food intake mice; RH: Reproducing high food intake mice; NL: Non-reproducing low food intake mice; RL: Reproducing low food intake mice. PC: Protein carbonyls; SOD: Superoxide dismutase; CAT: Catalase; GPx: Glutathione peroxidase. Different letters indicate significant differences between groups (post-hoc Tukey tests, p < 0.05).

**Table 5:** Descriptive statistics for oxidative markers measured in mammary tissue of lactating mice selected for high and low food intake.

Variables	RL	RH	Line
n	15	22	
PC	0.38 ± 0.03	0.37 ± 0.02	*F*_1,34_ = 0.07, *P* = 0.788
SOD	4.37 ± 0.55	4.20 ± 0.30	*F*_1,34_ = 0.09, *P* = 0.761
CAT	0.34 ± 0.04	0.29 ± 0.02	*F*_1,34_ = 0.83, *P* = 0.369
GPx	157.40 ± 11.60	153.48 ± 6.03	*F*_1,33_ = 0.11, *P* = 0.747

Measurements of various oxidative stress markers in mammary tissue of reproductive (R) mice from selection lines for high (H) or low (L) food intakes. Values shown are mean ± SEM. Results of one-way ANOVAs are shown with line and reproductive status as fixed factors. No significant interactions between line and reproductive status were found in the models and these were therefore removed before final analysis. RH: Reproducing high food intake mice; RL: Reproducing low food intake mice. PC: Protein carbonyls; SOD: Superoxide dismutase; CAT: Catalase; GPx: Glutathione peroxidase. Different letters indicate significant differences between reproductive statuses and lines.
