# Supplementary material for: Oxidative costs of reproduction in mouse strains selected for different levels of food intake and which differ in reproductive performance
Source: Sci Rep. 2016 Nov 14;6:36353. doi: 10.1038/srep36353 (PMC5107891; doi:10.1038/srep36353)
Supplement: Supplementary Information [file srep36353-s1.pdf]

Oxidative costs of reproduction in mouse strains selected for different levels of food intake and which differ in reproductive performance

Aqeel H. Al Jothery[1,2], Lobke M. Vaanholt[1], Nimesh Mody[3], Anis Amous[4], Jens Lykkesfeldt[4], Lutz Bünger[5], William G. Hill[6], Sharon E.Mitchell[1], David B. Allison[7]and John R. Speakman[1,8,*]

[1]Institute of Biological and Environmental Sciences, University of Aberdeen, Aberdeen AB24 2TZ, UK, [2]Department of Physiology and Pharmacology , College of Veterinary Medicine, University of Karbala, Karbala, Iraq, [3]Institute of Medical Sciences, University of Aberdeen, College of Life Sciences and Medicine, Foresterhill Health Campus, Aberdeen, United Kingdom,[4]Section of Experimental Animal Models, Faculty of Health & Medical Sciences,University of Copenhagen, Denmark, [5]Animal and Veterinary Science Group, Scotland's Rural College (SRUC), Edinburgh EH9 3JG, UK,[6]Institute of Evolutionary Biology, University of Edinburgh, Edinburgh EH9 3JT, UK,[7]School of Public Health, University of Alabama at Birmingham, Birmingham, Alabama, USA. [8]Institute of Genetics and Developmental Biology, State Key Laboratory of Molecular Developmental Biology, Chinese Academy of Sciences, Beijing, People's Republic of China,

[*]Author for correspondence (j.speakman@abdn.ac.uk

Materials and Methods

A-Reactive oxygen metabolites (ROMs) and Non-enzymatic antioxidant capacity (OXY)

The principle of ROMs assay is that the iron released from serum protein under an acid medium attacks hydroperoxide molecules present in serum and generate free radicals. These radicals react with chromogen to produce a stable coloured complex that can be measured by spectrophotometer. The serum and standard (10 μl each) were separately added to 1 ml of a reaction mixture containing 0.01 M acetetic acid/sodium acetate buffer (PH=4.8) and *N, N* -diethyl- *p*-phenylenediamine as chromogen, and then incubated for 90 min at 37°C. When the reactive oxygen metabolites react with chromogen, they produce a complex colour in which its intensity is directly proportional to their concentration. After incubation, the intensity of the colour was spectrophotometrically read at 505 nm at 37°C. The concentration of ROMs was calculated by dividing the absorbance of the sample and absorbance of the standard and multiplied by the concentration of standard. All samples, standards, and blanks were made in duplicate. The dROMS assay was expressed as mg H_2O_2/dL equivalents.

The OXY assay is based on the detecting the ability of non-enzymatic antioxidants to quench the oxidation effects of hypochlorous acid (HOCl). This assay has been previously used to measure total antioxidant capacity in different species[1–6] .The serum and standard were first diluted 1:100 with distilled water and then 10 μl from each of them was added to the 2ml eppendorf tube in duplicate. One millilitre of oxidant solution (HCIO based) was added to the blank, standard, and sample tubes and incubated for 10 min at 37°C. After incubation, the solution was poured into a cuvette already containing 10 μl of chromogen. The intensity of the colour was

inversely proportional to the concentration of non- enzymatic antioxidants capacity in which the change in the colour was spectrophotometrically read at 505 nm at 37°C. The concentration of OXY was calculated by using this formula OXY = (absorbance of blank-absorbance of sample)/(absorbance of blank-absorbance of standard)* standard. All samples, standards, and blanks were made in duplicate. The OXY was expressed as mM of HCIO.

B- Enzymatic antioxidant activities (CAT, SOD, and GPx)

Catalase activity is based on detecting the amount of H_2O_2 that was decomposed by catalase activity using $KMnO_4$. One hundred –eight microliters of supernatant was incubated with 18 μl of 10% Triton x100 on the ice for 30 minutes. For liver samples, the samples were diluted 1:20 with ice-cold 50Mm phosphate buffer before adding Triton x100. Sixty microliter of background sample (ice-cold 50Mm phosphate) and each sample was added to a 2 ml eppendorf tube in triplicate. Diluted H_2O_2 (6mM) was added to the tube and incubated on ice for 3 min. The reaction was stopped by adding H_2SO_4 (3M). Finally, $KmnO_4$ (2 mM) was added to the tube and absorbance was measured at 480nm. One unit of the enzyme activity was equivalent to k (U/min), where $k=\log (S_o/S_3) \times (2.3*V_t* DF)/ (V_S*T*X)$. S_o= difference between absorbance of standard and background, S_3=difference between absorbance of standard and sample, V_t=total volume of sample, H_2O_2, and H_2SO_4, DF=dilution factor, V_S= volumes of sample, T= incubation time, and X= amount of total protein (mg/ml).

The total SOD activity is based on the inhibition of the auto-oxidation of pyrogallol by the enzyme in the sample (with and without SOD) at 25°C and then followed kinetically at 420nm for 120 seconds with 2 seconds interval..The total mixture of reaction was constituted from 780 μl (50 mM Tris buffer), 10 μl supernatant of liver sample, and 10 μl of pyrogallol. For brain and mammary gland samples, 40 μl of their

supernatants and 10 μl of pyrogallol were added to the reaction mixture in a total volume of 800 μl. The measurement was firstly preceded by a blank, containing only 10 μl pyrogallol in 790 μl Tris buffer, and followed by measuring a mixture containing the sample in triplicate. One unit of SOD activity was expressed as the amount of the enzyme causing 50% inhibition of pyrogallol oxidation. SOD activity was expressed as U/mg protein, where SOD activity = (% inhibition*DF*V_t)/(50*V_S*X). DF=dilution factor, V_t=total volume of sample, VS= volume of sample, and X= amount of total protein (mg/ml).

The GPx activity is based on the oxidation of NADPH by GPx in the presence of reduced glutathione (GSH) and hyroperoxide at 25°C. The total mixture of reaction was constituted from 4.28 mM sodium azide (to inhibit catalase activity), 1.07 mM EDTA, 4.286 mM GSH, 0.214 mM NADPH, and 1 U/mL of glutathione reductase in ice-cold 50-mM phosphate buffer. Twenty-five microliters of H_2O_2 and a measured amount of sample, depending on the tissue (10, 25, and 50 μl in liver, mammary gland, and brain, respectively), were added to the reaction mixture. Reactions were followed kinetically at 340 nm for 60 seconds with 2 seconds interval in a total volume of 700 μl. The spontaneous NADPH oxidation reaction in the absence of enzyme was measured using a background (25 μl $H_2O_{2,}$ 10 μl of 50 mM Tris buffer, and 665 of reaction mixture) and then subtracted from the assay values. One unit of GPx was defined as the amount of enzyme that oxidized 1 mmol of NADPH per minute in the presence of reduced glutathione. Absorbance was read on a SPECTRA maxPlus microplate spectrophotometer (Molecular Devices Corp. Sunnyvale, CA, USA) and analysed using SOFT max Pro software (Molecular Devices Corp.). Background and samples were made in triplicate. GPx activity was expressed as nmole NADPH/ min/ mg protein, where GPx activity = (absorbance of sample/

min*Vt*DF)/ (0.00622*Vs*X). The activity of all enzymes was expressed per mg protein. Total protein content for different tissue samples was measured using the method of Bradford[7] . Protein content of the supernatant was measured using a Bradford assay (Quick Start Bradford protein essay kit 2; Biorad Laboratories, Hemel Hempstead, UK).

C-DNA damage (8OHdG) measured by ELISA and HPLC-ECD methods

For ELISA method, 100 mg of frozen liver tissue was homogenised with 1 ml of cold buffer A (10 mM Tris-HCL; 320 mM Sucrose; 5 Mm $MgCl_2$; 0.1 mM Deferoxamine mesylate; 1% TritonX-100). After centrifugation (4000g at 4°C for 5 minutes), the supernatant was discarded and the pellet was res-suspended in 1.5 ml cold buffer A. After second centrifugation (4000g at 4°C for 5 minutes), the pellet was suspended in 600 µl cold buffer B (10 mM Tris-HCL; 5 mM Na_2EDTA; 0.15 mM Deferoxamine mesylate). To exclude the RNA damage from being measured, the sample was incubated with (35µl of 10% SDS; 3µl RNase IIIA; 8µl RNase T1) at 50°C for 15 minutes. Thirty microliters of protease was then added and incubated at 37 °C for 1 hour. To extract the DNA from the sample, 2 ml of sodium iodide solution and 2 ml of absolute Isopropanol were added and followed by a centrifugation at 4000g at 4°C for 5 minutes. Supernatant was discarded and the pellet was washed twice by adding 1 ml of 40% Isopropanol and 1 ml of cold 70 % Ethanol, respectively, followed by a centrifugation at 4000g for 4°C for 5 minutes after each wash. To counteract the artefacts of oxidative damage associated with DNA isolation, extracted DNA was diluted with antioxidant (1 mM Deferoxamine mesylate). DNA quantification (µg/µl) in the sample was measured using the nanodrop (Molecular devices, Wokingham, UK). The ratio between two wavelengths 260/280 indicates the DNA quality, with 1.8 -1.9 values being for non-contaminated DNA. DNA samples (0.4 µg/µl) were

digested by adding 5µl (Nuclease P1) and incubated at 37 °C for 2.5 hours. Ten microliters of 500 mM Tris-base, (PH=8.3) and 5µl of Alkaline phosphatase were added and incubated at 37 °C for another 2.5 hours to further digest nucleotides to nucleosides. The amount of 8OHdG was detected using a competitive ELISA (Highly Sensitive 8-OHdG ELISA Kit, Japan Institute for the Control of Aging). Standards and blanks were made in triplicate while samples were made in duplicate. The DNA damage (8OHdG) was expressed as µg8OHdG/gDNA.

For HPLC-ECD method, DNA was prepared and oxidative damage (8OHdG) was measured using the protocol previously published with some modifications[8] . Concisely, 150-200 mg of frozen liver tissue was extracted in 1 ml cold buffer A (10 mM Tris buffer pH 7.5 containing 320mM sucrose, 5 mM $MgCl_2$, 1% Triton X-100, and 0.1 mM desferrioxamine). After centrifugation (10000g at 4°C for 30 seconds), the supernatant was discarded and the pellet was re-suspended in 180µl buffer B (40 mM Tris buffer pH 8 containing 20 mM Na_2EDTA, 7.6 mM NaI, and 0.1 mM desferrioxamine). The re-suspended pellet was then incubated with a mixture of 20 µl of SDS (10%), 30 µl RNase A, and 8 µl RNase T1 for 20 minutes at 50 °C. Protein of the sample was digested by adding 10µl proteinase K for one hour at 37°C. After centrifugation (10000g at 4°C for 5 minutes), 200 µl of the supernatant was transferred to 1.5 ml tube and incubated with 300 µl NaI 7.6 mM solution (20 mM Na_2EDTA, 40 mM Tris Base, and 0.1 mM desferrioxamine). DNA was then precipitated by mixing with 500 µl of concentrated 2-propanol and followed by centrifugation at 15000g for 3 minutes at 4 °C. The precipitated DNA was washed twice using 1 ml of 40% 2-propanol and followed by centrifugation at 15000g for 3 minutes at 4 °C. The final wash was performed by adding 1ml 70% ethanol which was subsequently evaporated under a stream of N_2 for 5 minutes. The dry DNA pellet

was diluted in 100 µl desferrioxamine /Tris buffer pH 7 (10mM Tris Base, 0.1 mM desferrioxamine). Hydrolysis of the extracted DNA was enzymatically performed using a mixture of nuclease P1 and alkaline phosphatase. Ten microliters of DOWEX resin (50µg/µl water) was added to the mixture for one minute and followed by centrifugation at 14000g for 5 minutes at 4 °C. Five hundred microliters the supernatant was transferred to a new tube and mixed with 50 µl of chloroform and followed by centrifugation at 10000g for 15 minutes at 4°C. Chromatographic separation of 8OHdG and dG was achieved on an Agilent Zorbax SB-Aq Narrow-Bore RR column (100 × 2.1 mm id; particle size 3.5 µm) protected by pre-column SecurityGard Ultra (Phenomenex: AJO-9000). The separation was performed under isocratic conditions with a mobile phase consisting of sodium phosphate 50 mM and sodium citrate 2.5 mM buffer (pH 7.0) containing 10% v/v methanol. The flow rate was set to 0.25 mL/min at 32°C and a total run time of the analysis was 25 min. Electrochemical detection was carried out using Dionex UltiMate 3000 HPLC electrochemical cell 6011 RS, with 8OHdG being detected at potential +300 mV while dG was detected at +800 mV. Prior to injection, samples were passed through 0.2µm RC filters (Phenomenex). Determination of 8OHdG was performed by injecting 10 µL while 1µL of diluted dG (10x times) was used for determination of dG. Standards and samples were made in duplicate and the damage expressed as a ratio between 8OHdG and 10^{*6} dG.

References

1. Costantini, D., Carello, L. & Fanfani, A. Relationships among oxidative status, breeding conditions and life-history traits in free-living Great Tits Parus major

and Common Starlings Sturnus vulgaris. *Ibis (Lond. 1859).* **152,** 793–802 (2010).

2. Markó, G., Costantini, D., Michl, G. & Török, J. Oxidative damage and plasma antioxidant capacity in relation to body size, age, male sexual traits and female reproductive performance in the collared flycatcher (Ficedula albicollis). *J. Comp. Physiol. B* **181,** 73–81 (2011).

3. Isaksson, C., Sheldon, B. C. & Uller, T. The Challenges of Integrating Oxidative Stress into Life-history Biology. *Bioscience* **61,** 194–202 (2011).

4. Beaulieu, M., Reichert, S., Le Maho, Y., Ancel, A. & Criscuolo, F. Oxidative status and telomere length in a long-lived bird facing a costly reproductive event. *Funct. Ecol.* **25,** 577–585 (2011).

5. Casagrande, S., Dell'omo, G., Costantini, D., Tagliavini, J. & Groothuis, T. Variation of a carotenoid-based trait in relation to oxidative stress and endocrine status during the breeding season in the Eurasian kestrel: a multi-factorial study. *Comp. Biochem. Physiol. Part A* **160,** 16–26 (2011).

6. Stier, A., Reichert, S., Massemin, S., Bize, P. & Criscuolo, F. Constraint and cost of oxidative stress on reproduction: correlative evidence in laboratory mice and review of the literature. *Front. Zool.* **9,** 37–48 (2012).

7. Bradford, M. M. A rapid and sensitive method for the quantitation of microgram quantities of protein utilizing the principle of protein-dye binding. *Anal. Biochem.* **72,** 248–254 (1976).

8. ESCODD. Measurement of DNA oxidation in human cells by chromatographic and enzymic methods. *Free Radic. Biol. Med.* **34,** 1089–1099 (2003).

Results

Supplementary Table 1-Effect of food intake (FI) and body mass (BM) on oxidative stress markers in various tissues of reproducing mice and non-reproducing female mice from high (H) and low (L) selection lines.

Factors		FI	Line	RS	RS×FI		BM	Line	RS	Line×BM	RS×BM	Line× RS×BM
Serum	*(df)*	*P*	*P*	*P*	*P*	*(df)*	*P*	*P*	*P*	*P*	*P*	*P*
dROMS	(1,50)	0.77	**<0.001**	**0.007**		(1,50)	0.60	**<0.001**	**<0.001**			
OXY	(1,50)	0.16	**0.007**	**0.01**		(1,50)	0.90	**0.02**	**0.03**			
OS index	(1,50)	0.32	**<0.001**	0.96		(1,50)	0.98	**<0.001**	0.14			
**Liver**												
PC	(1,36)	0.12	0.81	0.29		(1,36)	**0.03**	0.13	0.92			
8OHdG*	(1,16)	0.99	0.88	0.74	**0.02**	(1,17)	0.96	0.99	0.69			
8OHdG†	(1,20)	0.79	0.37	0.51		(1,20)	0.92	0.34	0.41			
SOD	(1,36)	0.87	0.64	0.96		(1,36)	0.06	0.72	0.87			
CAT	(1,36)	0.55	0.48	**0.03**		(1,36)	0.62	0.20	**0.002**			
GPx	(1,34)	0.63	**0.003**	**0.003**		(1,30)	0.14	0.17	0.55		**0.007**	**0.004**
**Brain**												
PC	(1,36)	0.71	0.66	0.16		(1,36)	0.18	0.84	**0.01**			
SOD	(1,35)	0.91	0.31	0.84		(1,34)	0.24	**0.05**	0.95	**0.03**		
CAT	(1,36)	0.56	0.78	0.26		(1,36)	0.38	0.91	**0.006**			
GPx	(1,31)	0.65	0.74	0.67		(1,31)	0.96	0.46	0.13			
**Mammary**												
PC	(1,33)	0.51	0.53			(1,33)	0.42	0.99				
SOD	(1,33)	0.76	0.96			(1,33)	0.43	0.60				
CAT	(1,33)	0.50	0.26			(1,33)	0.34	0.25				
GPx	(1,32)	**0.03**	0.25			(1,32)	0.12	0.83				

The table shows the results of general linear models with various biomarkers of serum oxidative stress (dROMS: Reactive oxygen metabolites; OXY: total non-enzymatic

antioxidants; OSI: Ratio between dROMS/ OXY *1000, PC: Protein carbonyls , *measured by ELISA method; † measured by HPLC method;; 8OHdG: 8-hydroxy-2-deoxyguanosine; SOD: Superoxide dismutase; CAT: Catalase; GPx: Glutathione peroxidase) as dependent factors, line (H vs. L) and reproductive status (RS) were added as fixed factors, and BM and FI were added to the models as covariate. Interaction terms were removed when not significance and the analyses repeated.

Supplementary Table 2-Effect of reproductive traits on oxidative stress markers in various tissues of reproducing mice and non-reproducing female mice from high (H) and low (L) selection lines.

**Factors**	**MEI**		**Line**	**Inter.**	**DEE**		**Line**	**Inter.**	**MEO**		**Line**	**Inter.**	**LS**		**Line**	**Inter**	**LM**		**Line**	**Inter.**
	df	*P*	*P*	*P*	*df*	*P*	*P*	*P*	*df*	*P*	*P*	*P*	*df*	*P*	*P*	*P*	*df*	*P*	*P*	*P*
dROMS	(1,31)	0.16	**0.06**		(1,31)	0.70	**0.001**		(1,31)	0.53	**0.05**		(1,31)	0.69	**0.001**		(1,31)	0.81	**0.02**	
OXY	(1,31)	0.66	0.15		(1,31)	0.47	0.27		(1,31)	0.41	**0.06**		(1,31)	0.10	**0.01**		(1,31)	0.35	**0.04**	
OSI	(1,31)	0.96	**0.01**		(1,31)	0.84	**0.002**		(1,31)	0.89	**0.009**		(1,31)	0.28	<**0.001**		(1,31)	0.44	**0.001**	
**Liver**																				
PC	(1,17)	0.12	0.50		(1,17)	0.40	0.80		(1,17)	0.14	0.55		(1,17)	0.06	0.91		(1,17)	**0.03**	0.29	
8OHdG*	(1,8)	0.48	0.73		(1,8)	0.42	0.97		(1,8)	0.69	0.97		(1,8)	0.32	0.93		(1,8)	0.40	0.69	
8OHdG†	(1,9)	0.46	0.99		(1,9)	0.24	0.07		(1,9)	0.21	0.80		(1,9)	0.85	0.23		(1,9)	0.62	0.70	
SOD	(1,16)	0.09	**0.02**	**0.02**	(1,17)	0.77	0.53		(1,16)	0.10	**0.01**	**0.01**	(1,17)	0.51	0.60		(1,17)	0.59	0.84	
CAT	(1,17)	0.51	0.48		(1,17)	0.83	0.85		(1,17)	0.40	0.40		(1,17)	0.66	0.65		(1,17)	0.28	0.31	
GPx	(1,17)	0.22	0.36	**0.03**	(1,16)	0.48	<**0.001**		(1,16)	0.95	**0.001**		(1,16)	0.85	<**0.001**		(1,16)	0.36	**0.002**	
**Brain**																				
PC	(1,17)	0.22	0.84		(1,17)	0.24	0.71		(1,17)	0.33	0.99		(1,17)	0.59	0.44		(1,17)	0.50	0.84	
SOD	(1,17)	0.95	0.72		(1,17)	0.70	0.47		(1,17)	0.83	0.80		(1,17)	0.75	0.64		(1,17)	0.26	0.68	

CAT	(1,17) 0.76 0.67	(1,17) 0.81 0.46	(1,17) 0.79 0.64	(1,17) 0.56 0.47	(1,17) 0.39 0.97
GPx	(1,15) 0.96 0.94	(1,14) 0.06 0.09 **0.04**	(1,15) 0.86 0.93	(1,15) 0.69 0.85	(1,15) 0.69 0.80
**Mammary tissue**					
PC	(1,33) 0.27 0.30	(1,33) 0.26 0.37	(1,33) 0.44 0.45	(1,33) 0.19 0.45	(1,33) 0.47 0.48
SOD	(1,33) 0.61 0.83	(1,33) 0.12 0.23	(1,33) 0.21 0.47	(1,32) 0.82 **0.03**	(1,33) 0.85 0.73
				**0.03**	
CAT	(1,33) 0.34 0.19	(1,33) 0.68 0.66	(1,33) 0.20 0.12	(1,33) 0.93 0.39	(1,33) 0.83 0.65
GPx	(1,32) 0.37 0.61	(1,32) 0.89 0.88	(1,32) 0.31 0.57	(1,32) 0.30 0.89	(1,32) 0.38 0.67

The table shows the results of general linear models with various biomarkers of serum oxidative stress (dROMS: Reactive oxygen metabolites; OXY: total non-enzymatic antioxidants; OSI: Ratio between dROMS/ OXY *1000, PC: Protein carbonyls , *measured by ELISA method; † measured by HPLC method;; 8OHdG: 8-hydroxy-2-deoxyguanosine; SOD: Superoxide dismutase; CAT: Catalase; GPx: Glutathione peroxidase) measured in various tissues as dependent factors and line (H vs. L) as fixed factors, and reproductive traits (MEI: Metabolisable energy intake; DEE: Daily energy intake; MEO: milk energy output; LS: litter size; LM: litter mass) were added to the models as covariate. Interaction terms were removed when not significance and the analyses repeated.
